# 1237. Characterization and crystallization of OXA-935, a novel class D OXA-10-like beta-lactamase, found in *Pseudomonas aeruginosa*

**DOI:** 10.1093/ofid/ofab466.1429

**Published:** 2021-12-04

**Authors:** Nathan B Pincus, Monica Rosas-Lemus, Samuel W Gatesy, Ludmilla Shuvalova, George Minasov, Karla Satchell, Joseph S Brunzelle, Marine Lebrun-Corbin, Egon A Ozer, Alan R Hauser, Kelly E R Bachta

**Affiliations:** 1 Northwestern University, Chicago, IL; 2 Northwestern University Feinberg School of Medicine, Chicago, Illinois; 3 Northwestern University, Feinberg School of Medicine, Chicago, Illinois

## Abstract

**Background:**

Recently, we described a collection of ST298 *Pseudomonas aeruginosa* (PA) isolates that caused a prolonged epidemic of XDR infections. Many of these contain derivatives of a new plasmid, pPABL048, that harbors an MDR integron, in1697. In1697 contains a series of antimicrobial resistance (AMR) genes, one of which is the class D β-lactamase *bla*_OXA-10_. Variants of *bla*_OXA-10_ have been described that confer both extended-spectrum β-lactamase (ESBL) and carbapenemase activity.

**Methods:**

Of all ST298 isolates, three were resistant to ceftazidime (CTZ). Genomic comparison of in1697 in CTZ-resistant and CTZ-sensitive strains revealed that all three strains harbored a *bla*_OXA-10_ allele with two single nucleotide variations resulting in amino acid changes at positions 153 (F153S) and 157 (G157D). Using the NCBI database, we identified this allele as unique and defined this β-lactamase as OXA-935. OXA-935 shares the G157D variation with OXA-14 which is known to confer resistance to ceftazidime. We sought to characterize the function of OXA-935 and to determine the crystal structures of OXA-14 and OXA-935.

**Results:**

Deletion of *bla*_OXA-935_ phenotypically converted all three strains to CTZ-susceptible. Expression of *bla*_OXA-14_ and *bla*_OXA-935_ conferred CTZ-resistance to laboratory PA strains PA01 and PA14. Determination of the crystal structures of OXA-14 (PDB code 7L5R) and OXA-935 (PDB code 7L5V) revealed that the F153S variant resulted in increased flexibility in the enzyme’s Ω loop. Conformational changes in the Ω loop likely contributed to the lack of carbamylation at lysine-70 (K70) observed in OXA-935. Carbamylation of K70 is known to be critical for enzymatic activity of class D β-lactamases.

**Conclusion:**

OXA-935 is very similar to OXA-14; however, comparison revealed that the F153S variant has unique structural features and is functionally distinct. Despite these differences, both enzymes confer high-level CTZ resistance. As we increasingly rely on β-lactam antimicrobial therapy (e.g. ceftazidime, cefepime) and combination (e.g. ceftazidime-avibactam) therapy to treat MDR PA infections, it is critical that we continue to explore the mechanistic basis of β-lactam AMR in an effort to preserve existing treatments and design novel ones.

**Disclosures:**

**All Authors**: No reported disclosures

